# Fabrication of Vertical Array CNTs/Polyaniline Composite Membranes by Microwave-Assisted In Situ Polymerization

**DOI:** 10.1186/s11671-015-1201-z

**Published:** 2015-12-24

**Authors:** Jie Ding, Xiaoyan Li, Xia Wang, Jinrui Zhang, Dengguang Yu, Biwei Qiu

**Affiliations:** School of Material Science and Engineering, University of Shanghai for Science and Technology, Shanghai, 200093 China; School of Environment and Architecture, University of Shanghai for Science and Technology, Shanghai, 200093 China

**Keywords:** Vertical array carbon nanotubes, Polyaniline, Composite, Microwave assisted

## Abstract

A vertical array carbon nanotubes (VACNTs)/polyaniline (PANi) composite membrane was prepared by microwave-assisted in situ polymerization. With microwave assistance, the morphology of PANi revealed a smaller diameter and denser connection. Meanwhile, thermogravimetric analysis showed improved thermal stability of microwave-assisted PANi for higher molecular weight. Focused ion beam thinning method was used to cut the VACNTs/PANi membrane into dozen-nanometer thin strips along the cross-sectional direction, and transmission electron microscopy observation showed seamless deposition of PANi between VACNT gaps, without damaging the vertical status of CNTs. Meanwhile, stronger conjugate interaction between the quinoid ring of PANi and VACNTs of the composite membrane were prompted by microwave-assisted in situ polymerization. By using nanoindentation technology, the VACNTs/PANi composite membrane showed exponential increasing of modulus and hardness. Meanwhile, the elasticity was also improved, which was proved by the calculated plastic index. The results can provide helpful guidance for seamlessly infiltrating matrix into CNT array and also demonstrate the importance of structural hierarchy for getting proper behavior of nanostructures.

## Background

Carbon nanotubes (CNTs) [1], with graphene composed of carbon atoms curling to a hollow tubular structure, possess an ultra-high aspect ratio, atomically smooth nanoscale pores for gas transport [2, 3], high mechanical strength [4], and unique electronic properties [5–7]. Vertical array CNT (VACNT) membranes have found many promising applications such as being supercapacitors [8], being compliant thermal interface materials [9], in selective gas transport [10], and being reinforcements in composites for enhanced thermal and mechanical properties [11].

Infiltrating a matrix material into a large-surface-area VACNT membrane has attracted great attention for obtaining a novel composite membrane with synergic properties and improved performances. Several routes, such as chemical vapor deposition (CVD) [12] and spin coating, can be used to deposit a matrix into the space between vertically aligned and dense carbon-packed CNTs. Hinds et al. [13] grew well-aligned multi-walled carbon nanotubes (MWCNTs) via CVD; then, a 50 wt% solution of polystyrene and toluene was spin-coated over the surface. However, spin coating may destroy CNT arrays because of its centrifugal force. Holt et al. [14] presented an approach for depositing silicon nitride into a MWCNT array via a low-pressure CVD method; then, the tubes were etched to prepare a seamless composite membrane. This method could successfully avoid destroying CNT arrays; however, silicon nitride was too brittle which limited the mechanical properties of the membrane. Miserendino [15] and Zhang [16] filled in the gaps of MWCNTs with parylene matrix via CVD and found that parylene could fully pad CNT gaps. Polymer materials exhibit good chemical stability and biocompatibility, easily filling implement, while choosing the appropriate polymer into VACNTs is a considerable challenge.

Polyaniline (PANi) is a widely studied low-cost electrically conducting polymer that exhibits facile synthesis and environmental stability [17, 18]. Over the last decades, the chemical synthesis approach has been used to obtain a wide variety of PANi with different morphologies and properties. Ramana [19] prepared a PANi-coated CNT composite thin film via an in situ rapid mixing chemical oxidative polymerization method. Polysulfone composite membranes created with PANi and functionalized multi-walled CNTs were synthesized using a non-solvent/solvent-induced phase separation technique in our research group [20]. In addition, MWCNTs/PANi composite membranes were successfully fabricated by filtration and the flash welding method in our previous work [21]. PANi can be synthesized through different approaches to obtain composites with other materials, such as in situ polymerization [22–25], electrochemical processes [26], microemulsion polymerization [27], and interfacial polymerization [28]. As a novel energy, microwave can quickly absorb electromagnetic energy and generate rapid thermal effects through molecular dipole interaction [29]. Microwave-assisted synthesis can be an effective strategy to control the structure and morphology of a polymer. A PANi nanofiber-coated graphite electrode was successfully fabricated by microwave-assisted chemical vapor-induced in situ polymerization, with mixed structures of emeraldine base and fully oxidized form [30].

In this study, PANi was deposited seamlessly into the space between VACNTs by microwave-assisted in situ polymerization. The structure and thermal stability of PANi were characterized by Fourier transform infrared (FTIR) spectroscopy and thermogravimetric analysis (TGA), respectively. Scanning electron microscopy (SEM) and transmission electron microscopy (TEM) were used to observe the morphology of PANi and VACNTs/PANi composite membrane. The structure and interaction of PANi and VACNTs were further explained by FTIR and Raman spectroscopy. In addition, the nanoscale mechanical properties of the VACNTs/PANi membrane were discussed according to the nanoindention measurement. The results of the investigation revealed that microwave could be an effective way for in situ polymerization and impregnation of PANi between the VACNT nanotube gaps.

## Methods

### Materials

The VACNTs (MWNT array with 3–10-nm diameter and 50-μm length, 98 wt%) were purchased from Chengdu Organic Chemical Co., Ltd., Chinese Academy of Sciences (Chengdu, China). Aniline (ANi) as the monomer, ammonium persulfate (APS) as the catalyst, and hydrochloric acid (HCl) (AR grade) were purchased from Sinopharm Chemical Reagent Co., Ltd. (Shanghai, China).

### Synthetic Procedures

**Fig. 1:**
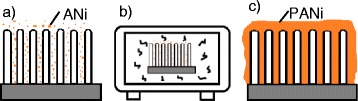
Schematic of the fabrication process. **a** Monomer aniline immersed into VACNT gaps. **b** Microwave irradiation. **c** VACNTs/PANi composite

PANi was prepared via the chemical oxidization of aniline with APS. Appropriate amounts of aniline (6 mmol) and APS (1.5 mmol) were dissolved in 1 vol% HCl (20 ml) solution separately (the volume ratio of aniline was about 5 %). The proper proportion of aniline and APS was chosen as 4:1 based on previous experiments, according to the denser and uniform morphology. The commercial VACNTs (1 cm × 1 cm) were placed vertically in precursor aniline solution for 1 h to let aniline absorb on the wall of VACNTs. APS solution was slowly added to the above mixture while stirring. The glass beaker that contained the precursor solution and the VACNT substrate was placed in a water bath, then they were put in the center of a microwave oven (MCR-3, Gongyi Yuhua Instrument Co., Ltd., China), and the microwave oven was set with a temperature mode of 50 °C and radiation time of 15 min. The procedure was repeated for eight times, and the total microwave irradiation time was 120 min, the schematic of the fabrication process was shown in Fig. 1. Afterwards, the prepared VACNTs/PANi membrane was washed with water and then freeze-dried. Meanwhile, the powder of the suspension was also collected by filtration for characterizations.

To observe surface morphology easily, we prepared PANi on a cellulose membrane using the same synthesis process, and the product was coded as PANi-MW (powder or film, depending on the ways of sample collection). In addition, PANi polymerized by chemical oxidation at room temperature without microwave irradiation was synthesized for comparison, and the product was also coded as PANi (powder or film).

### Characterizations

The structures of PANi and VACNTs/PANi composite were characterized by FTIR (Spectrum 100, PerkinElmer Co., Ltd., USA) and Raman (LabRam-1B, Horiba Jobin Yvon Co., Ltd., USA, laser 532 nm) spectroscopy, respectively. Thermal stability was characterized by TGA (Pyris 1, PerkinElmer Co., Ltd., USA) in nitrogen atmosphere with a 10 °C/min heating rate from 50 to 800 °C. The molecules of PANi were measured via gel permeation chromatography (GPC 50, Waters Co., Ltd., USA) and dissolved in *N*,*N*-dimethylformamide. Morphology characterizations were imaged using SEM (Quanta FEG450, FEI Co., Ltd., USA) and TEM (Tecnai G2 20 TWIN, FEI Co., Ltd., USA). Focused ion beam (FIB, Helios Nanolab 600, FEI Co., Ltd., USA) was used to prepare the samples with a thickness of approximately 70 nm for TEM observation. Nanoindentation (Agilent Nano G200) was performed to investigate the nanomechanical properties of the samples.

## Results and Discussion

### FTIR Spectroscopy of PANi

**Fig. 2:**
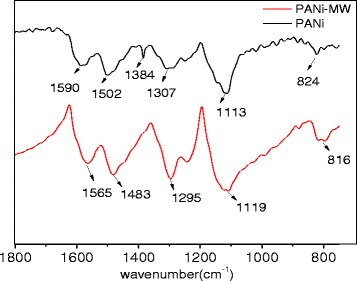
FTIR spectra of PANi and PANi-MW

The FTIR spectra of the PANi samples obtained under different synthesis conditions are shown in Fig. 2. PANi exhibited typical characteristic bands at 1590 and 1502 cm^−1^, attributed to the quinoid and benzenoid structure, respectively. The spectra of PANi-MW were roughly the same as those of PANi, but differed in two peaks. The quinoid band and benzenoid band revealed a red shift from 1590 and 1502 cm^−1^ to 1565 and 1483 cm^−1^, respectively, which indicated that microwave irradiation could enhance the conjugated effect for PANi [31]. Moreover, the band at 1384 cm^−1^ disappeared for the electron clouds were shared in N atoms instead of “pocketed” to form conjugated systems via microwave-assisted method, and the conjugation systems were further enhanced.

### Raman Spectroscopy of PANi

**Fig. 3:**
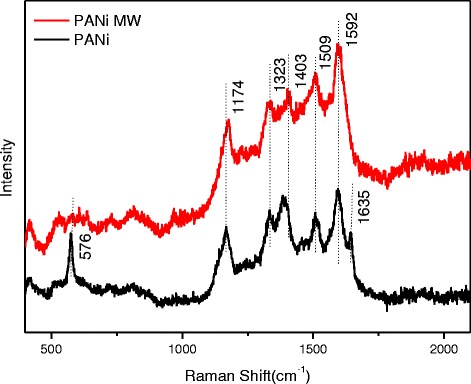
Raman spectra of PANi and PANi-MW

Raman spectra obtained from the samples are shown in Fig. 3. PANi were identified with typical peaks of 1635 cm^−1^ (C-C benzenoid), 1592 cm^−1^ (C=C quinoid), 1509 cm^−1^ (C=N quinoid), 1403 cm^−1^ (Ar-N benzenoid), 1323 cm^−1^ (C-N benzenoid), 1174 cm^−1^ (C-H), and 576 cm^−1^ (in-plane benzenoid ring deformation). With microwave assistance, the characteristic band of PANi-MW showed to be similar but differed a little. The quinoid relevant bands showed to be stronger while the benzenoid bands showed to be weaker. Meanwhile, C-C (1635 cm^−1^) and in-plane benzenoid ring deformation (576 cm^−1^) disappeared, indicating that the microwave irradiation provided the energy for increasing the quinoid fraction.

### Surface Morphology of PANi

**Fig. 4:**
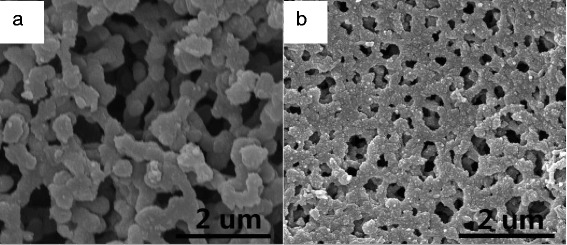
SEM images of **a** PANi and **b** PANi-MW on a cellulose membrane

The surface morphologies of PANi-MW and PANi synthesized at room temperature (deposited on a cellulose membrane) are shown in Fig. 4. The PANi prepared at room temperature presented nanorod shapes, diameters ranging from 300 to 500 nm. PANi-MW morphology revealed a smaller diameter and denser connection states than that of PANi. PANi-MW was nearly completely covered with less overgrown granules that resulted from the uniform energy reception of microwave radiation. A dense topography of PANi-MW was favorable for seamlessly filling in the CNT array.

### Molecular Measurements

The number average molecular mass (*M*_n_) and polydispersity index (PDI) of PANi and PANi-MW are shown in Table 1. The *M*_n_ of PANi-MW was 5.09 × 10^5^, nearly five times that of PANi (*M*_n_ = 1.08 × 10^5^). Meanwhile, the PDI of PANi-MW was narrower than that of PANi. GPC analysis proved more homogeneous and longer PANi molecular chains can be generated by microwave-assisted in situ polymerization.

**Table 1:** GPC analysis of PANi and PANi-MW

Sample	*M*_n_	PDI
PANi	1.08 × 10^5^	3.64
PANi-MW	5.09 × 10^5^	1.62

### TG Analysis

**Fig. 5:**
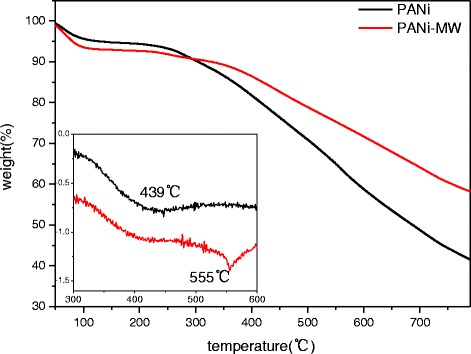
TG spectra of PANi and PANi-MW in nitrogen

The TG result of PANi is shown in Fig. 5. The weight loss below 100 °C could be assigned to the loss of the initial water molecules. Two weight loss stages were observed after 100 °C. From 100 to 300 °C, weight loss could be mainly attributed to the decomposition of PANi with low molecular weight. Rapid weight loss occurred in the temperature range of 300 to 600 °C, and the peak decomposition temperature of PANi-MW was improved from 439 °C (PANi) to 555 °C, as seen in inset of Fig. 5; in addition, the residuals (at 600 °C) of PANi-MW were increased from 58.3 % (PANi) to 71.2 %. The improved thermostability could be attributed to the more conjugated structure of PANi-MW caused by microwave irradiation, and the result was consistent with the GPC analysis for the higher molecular and narrower PDI of PANi-MW.

### SEM of VACNTs/PANi Composite

**Fig. 6:**
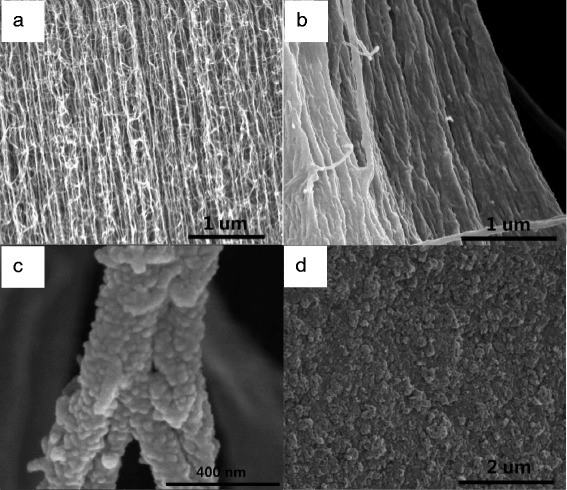
SEM images of **a** raw VACNTs side section, **b** VACNTs/PANi composite side section, **c** enlarged PANi-coated CNTs, and **d** VACNTs/PANi surface

Compared to the raw VACNTs in Fig. 6a, seamless and complete polymer coating is shown in Fig. 6b. In addition, the vertical array structure was unaffected by the microwave-assisted in situ polymerization, exhibited by the “standing” forest morphology in the side section of the VACNTs/PANi composite. As shown in the high-magnification image (Fig. 6c), the diameter of VACNTs was significantly larger, coated uniformly and completely with rough PANi nanoparticles. Meanwhile, no large void or crack was observed on the surface of the VACNTs/PANi composite membrane (Fig. 6d).

### TEM of VACNTs/PANi Cross Section

To further reveal the PANi-occupied state between VACNT gaps, the cross section of the VACNTs and VACNTs/PANi composite membrane was analyzed by TEM observation.

**Fig. 7:**
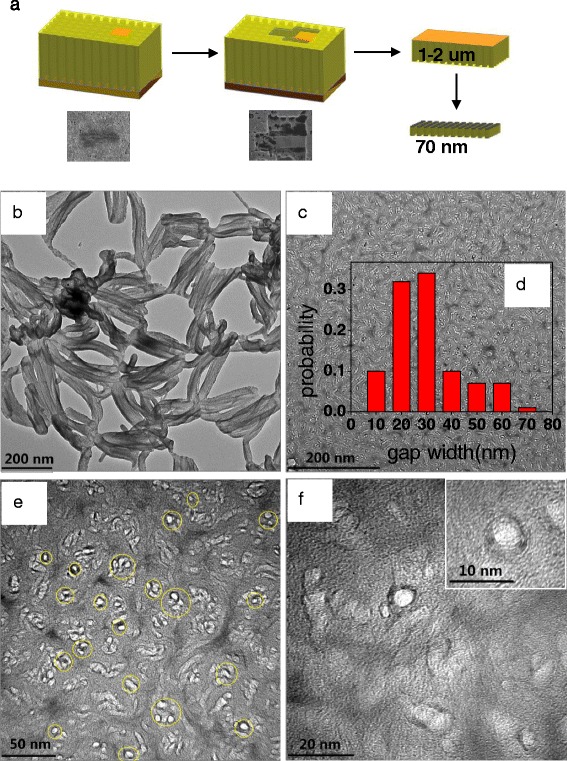
FIB sample preparation and TEM images. **a** TEM sample prepared by FIB. **b** TEM images of VACNT cross section. **c**, **e**, **f** TEM images of VACNTs/PANi composite membrane cross section at different magnifications. **d** Histogram of gap width between the tubes

In this paper, the TEM cross section sample was prepared by FIB method, shown in Fig. 7a. The FIB slicing processing is as follows. First, an area (approximately 10 μm) was selected, and a protective layer of platinum was deposited on the surface. Second, three sides of the sample were slotted, and then the bottom was cut, followed by cutting off the right side after adhering the sample to the tungsten probe. Third, the sample was conglutinated to the special copper mesh. Finally, the sample was cut to a desired thickness (approximately 70 nm) by FIB.

As shown in Fig. 7b, the cross section of VACNTs was broken with loose nanotube distribution. FIB processing may lead to bundling phenomenon of VACNTs, showing a much bigger diameter. TEM morphology of the VACNTs/PANi cross section (Fig. 7c) showed continuous PANi distribution between the nanotube gaps, providing good support effect. The bright white spots in Fig. 7e are carbon nanotube pores, and the multiwall and the surrounding polymer structures could be observed in Fig. 7f and inset. The TEM images demonstrated that PANi coated VACNTs properly and did not leave any gap and cracks, and the nanotubes kept the vertical state during the FIB slicing process. The average gap width between the nanotubes was estimated to be 30 ± 5 nm, which could be derived from the histogram of the gap width between the nanotubes (counted from 324 individual CNTs) in Fig. 7d.

### Structure of VACNTs/PANi Composite

**Fig. 8:**
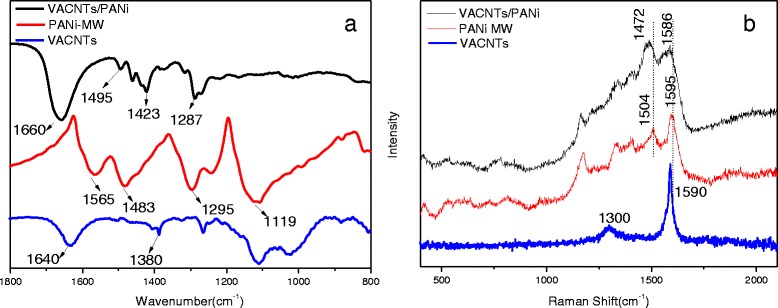
**a** FTIR and **b** Raman spectra for VACNTs, PANi-MW, and VACNTs/PANi composite

FTIR and Raman spectra of VACNTs, PANi-MW, and the VACNTs/PANi composite are shown in Fig. 8 to confirm the structures. In Fig. 8a, the characteristic bands of 1640 and 1380 cm^−1^ indicated the C=C of VACNTs. In the VACNTs/PANi composite, it was noticed that there was a single band of 1660 cm^−1^ for mergence of the quinoid band (1565 cm^−1^ in PANi-MW) and C=C band (1640 cm^−1^ in VACNTs). Meanwhile, with the presence of VACNTs, the PANi structure was influenced with a more red shift of C-N (1295 to 1287 cm^−1^), indicating a strong interaction between VACNTs and PANi via π-stacking [32]. Moreover, strengthened quinoid (1423 cm^−1^) and disappeared N=quinoid=N (1119 cm^−1^) in the VACNTs/PANi composite revealed the conjugate interaction between the quinoid unit of PANi and VACNTs.

As for the VACNTs, the characteristic Raman bands at ~1595 cm^−1^ (G band) and ~1300 cm^−1^ (D band) indicated graphitic nature and disorderness pertaining to VACNTs, respectively. The G band to D band intensity ratio of approximately 6.32 indicated the high crystallinity of the MWCNTs. However, in the case of the VACNTs/PANi composite, the disappearance of the characteristic G band indicated that the VACNTs were coated with PANi, while the vibration peak at 1472 cm^−1^ increased with VACNTs. Meanwhile, there was a considerable red shift in characteristic bands corresponding to C=C (from 1595 to 1586 cm^−1^) and C=N (from 1504 to 1472 cm^−1^) stretching of quinoid, consistent with the FTIR results. VACNTs were electron-rich molecules that form π-π interaction and CH-π interaction, and microwave irradiation offered the energy for the formation of a charge-transfer complex between the VACNTs and aniline. Aromatic structures, in general, were known to interact strongly with the basal plane of graphitic surface via π-stacking which was due to a charge transfer from the quinoid unit of PANi to VACNTs.

### Nanoindentation of VACNTs/PANi Composite

**Fig. 9:**
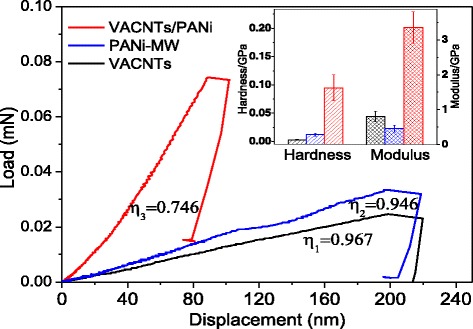
Nanoindention of VACNTs/PANi, VACNTs, and PANi-MW

Nanoindentations of raw VACNTs, PANi-MW, and the VACNTs/PANi composite were performed to evaluate the PANi deposition effect for VACNTs. For comparison, PANi-MW was deposited on the silicon substrate, the same as that of raw VACNTs and the VACNTs/PANi composite membrane (Fig. 9).

The modulus and hardness of VACNTs/PANi were 3.354 ± 0.450 and 0.095 ± 0.0230 GPa, respectively, which improved more than four times in modulus compared with that of raw VACNTs and seven times compared with that of PANi, as seen in inset of Fig. 9. After PANi was deposited seamlessly into the gaps between VACNTs, the reinforcement effect on the composite membrane was evident. In addition, the plastic indexes (marked as *η* in Fig. 9), which were used to measure the relative plastic deformation during the indentation, were calculated according to the three nanoindentation curves. From the calculation, *η*_3_ (VACNTs/PANi) was 0.756, which reduced 23 and 21 % compared with that of VACNTs and PANi-MW, respectively. The PANi acted as elastic links between the CNTs and increased the elastic recovery upon unloading. Therefore, VACNTs/PANi recovered a greater portion of the compressed length and absorbed a greater amount of energy. Seamless polymerization of PANi in VACNT gaps and stronger interaction effect can make up deficiencies of VACNTs in array stability, stiffness, and elastic recovery, so as to broaden the fields of applications, such as selective transport membrane and micro-electromechanical devices.

## Conclusions

In summary, an effective method to polymerize PANi in situ seamlessly into VACNTs was successfully developed by microwave-assisted chemical bath deposition method. With microwave irradiation, an enhanced conjugate with a more quinoid structure of PANi could be found; a smaller diameter and denser connection morphology together with a higher molecular weight and narrower PDI could be realized, showing a better thermal stability. The morphology of VACNTs/PANi presented a uniform and seamless PANi deposition between VACNT gaps, and VACNTs maintained a highly aligned forest structure. Confirmed by FTIR and Raman spectra, the highly conjugated π-stacking between PANi and VACNTs are prompted by microwave-assisted in situ polymerization. Nanoindentation showed a significant improvement in modulus and hardness for the VACNTs/PANi membrane. In addition, the elasticity of VACNTs/PANi was improved in a remarkable degree for plastic index decreasing over 20 %, which can be attributed to seamlessly filling of PANi in the VACNTs and strong interaction effect between PANi and CNTs. The obtained results in this study will enhance the ability to design and validate the performance of the VACNTs/polymer composite membrane and its microstructure for many applications.

## References

[1] Iijima S (1991). Helical microtubules of graphitic carbon. Nature.

[2] López-Lorente AI, Simonet BM, Valcárcel M (2010). The potential of carbon nanotube membranes for analytical separations. Anal Chem.

[3] Zhao B, Futaba DN, Yasuda S (2009). Exploring advantages of diverse carbon nanotube forests with tailored structures synthesized by supergrowth from engineered catalysts. ACS Nano.

[4] Muhsan AS, Ahmad F, Mohamed NM (2014) Effect of CNTs dispersion on the thermal and mechanical properties of Cu/CNTs nanocomposites. AIP Conf Proc 1621:643–649

[5] Srivastava A, Jain N, Nagawat AK (2013). Effect of Stone-Wales defects on electronic properties of CNTs: ab-initio study. Quantum Matter.

[6] Rafiee R, Sabour MH, Nikfarjam A (2014). The influence of CNT contents on the electrical and electromagnetic properties of CNT/Vinylester. J Electron Mater.

[7] Yardimci AI, Tanoglu M, Selamet Y (2013). Development of electrically conductive and anisotropic gel-coat systems using CNTs. Prog Org Coat.

[8] Youn-Su K, Kitu K, Fisher FT (2012). Out-of-plane growth of CNTs on graphene for supercapacitor applications. Nanotechnology.

[9] Fabris D, Rosshirt M, Cardenas C (2011). Application of carbon nanotubes to thermal interface materials. J Electron Packag.

[10] Yang HY, Han ZJ, Yu SF (2013). Carbon nanotube membranes with ultrahigh specific adsorption capacity for water desalination and purification. Nat Commun.

[11] Amr IT, Al-Amer A, Selvin TP (2011). Effect of acid treated carbon nanotubes on mechanical, rheological and thermal properties of polystyrene nanocomposites. Compos Part B.

[12] Zhao N, He C, Li J (2006). Study on purification and tip-opening of CNTs fabricated by CVD. Mater Res Bull.

[13] Hinds BJ, Chopra N, Rantell T (2004). Aligned multiwalled carbon nanotube membranes. Science.

[14] Holt JK, Park HGY, Wang YM (2006). Fast mass transport through sub-2-nanometer carbon nanotubes. Science.

[15] Scott M, Juhwan Y, Alan C (2006). Electrochemical characterization of parylene-embedded carbon nanotube nanoelectrode arrays. Nanotechnology.

[16] Zhang L, Yang J, Wang X (2014). Temperature-dependent gas transport performance of vertically aligned carbon nanotube/parylene composite membranes. Nanoscale Res Lett.

[17] Li D, Huang J, Kaner RB (2008). Polyaniline nanofibers: a unique polymer nanostructure for versatile applications. Acc Chem Res.

[18] Tran HD, Li D, Kaner RB (2009). 1D conducting polymer nanostructures: one-dimensional conducting polymer nanostructures. Adv Mater.

[19] Ramana GV, Padya B, Srikanth VVSS (2014). Rapid mixing chemical oxidative polymerization: an easy route to prepare PANI coated small-diameter CNTs/PANI nanofibres composite thin film. Bull Mater Sci.

[20] Liao YZ, Yu DG, Wang X (2013). Carbon nanotube-templated polyaniline nanofibers: synthesis, flash welding and ultrafiltration membranes. Nanoscale.

[21] Cai SS, Li XY, Wang X (2014). Preparation of MWNTs/polyaniline composite membranes by filtration and flash welding method. Indian J Eng Mater Sci.

[22] Wei Z, Wan M, Lin T (2003). Polyaniline nanotubes doped with sulfonated carbon nanotubes made via a self‐assembly process. Adv Mater.

[23] Small WR, Masdarolomoor F, Wallace GG (2007). Inkjet deposition and characterization of transparent conducting electroactive polyaniline composite films with a high carbon nanotube loading fraction. J Mater Chem.

[24] Ginic-Markovic M, Matisons JG, Cervini R (2006). Synthesis of new polyaniline/nanotube composites using ultrasonically initiated emulsion polymerization. Chem Mater.

[25] Salvatierra RV, Oliveira MM, Zarbin AJG (2010). One-pot synthesis and processing of transparent, conducting, and freestanding carbon nanotubes/polyaniline composite films. Chem Mater.

[26] Bhadra S, Singha NK, Khastgir D (2007). Electrochemical synthesis of polyaniline and its comparison with chemically synthesized polyaniline. J Appl Polym Sci.

[27] Prasannan A, Somanathan N, Hong PD (2009). Studies on polyaniline–polypyrrole copolymer micro emulsions. Mater Chem Phys.

[28] Abdolahi A, Hamzah E, Ibrahim Z (2012). Synthesis of uniform polyaniline nanofibers through interfacial polymerization. Materials.

[29] Wiesbrock F, Hoogenboom R, Schubert US (2004). Microwave-assisted polymer synthesis: state-of-the-art and future perspectives. Macromol Rapid Commun.

[30] Li XQ, Yang L, Lei Y, Gu L, Xiao D (2014). Microwave-assisted chemical-vapor-induced in situ polymerization of polyaniline nanofibers on graphite electrode for high-performance supercapacitor. ACS Appl Mater Interfaces.

[31] Gizdavic-Nikolaidis MR, Jevremovic MM, Allison MC (2014). Self-assembly of nanostructures obtained in a microwave-assisted oxidative polymerization of aniline. Express Polym Lett.

[32] Mi HY, Zhang XG, An SY, Ye XG, Yang SD (2007). Microwave-assisted synthesis and electrochemical capacitance of polyaniline/multi-wall carbon nanotubes composite. Electrochem Commun.

